# The role of apoptosis in spinal cord injury: a bibliometric analysis from 1994 to 2023

**DOI:** 10.3389/fncel.2023.1334092

**Published:** 2024-01-16

**Authors:** Siqiao Wang, Liming Cheng

**Affiliations:** ^1^Division of Spine, Department of Orthopedics, Tongji Hospital Affiliated to Tongji University School of Medicine, Shanghai, China; ^2^Key Laboratory of Spine and Spinal Cord Injury Repair and Regeneration (Tongji University), Ministry of Education, Shanghai, China; ^3^Institute of Spinal and Spinal Cord Injury, Tongji University School of Medicine, Shanghai, China; ^4^Stem Cell Translational Research Center, Tongji Hospital, Tongji University School of Medicine, Shanghai, China

**Keywords:** apoptosis, spinal cord injury, inflammation, activation, functional recovery, Bibliometrix

## Abstract

**Background:**

Apoptosis after spinal cord injury (SCI) plays a pivotal role in the secondary injury mechanisms, which cause the ultimate neurologic insults. A better understanding of the molecular and cellular basis of apoptosis in SCI allows for improved glial and neuronal survival via the administrations of anti-apoptotic biomarkers. The knowledge structure, development trends, and research hotspots of apoptosis and SCI have not yet been systematically investigated.

**Methods:**

Articles and reviews on apoptosis and SCI, published from 1st January 1994 to 1st Oct 2023, were retrieved from the Web of Science™. Bibliometrix in R was used to evaluate annual publications, countries, affiliations, authors, sources, documents, key words, and hot topics.

**Results:**

A total of 3,359 publications in accordance with the criterions were obtained, which exhibited an ascending trend in annual publications. The most productive countries were the USA and China. *Journal of Neurotrauma* was the most impactive journal; Wenzhou Medical University was the most prolific affiliation; Cuzzocrea S was the most productive and influential author. “Apoptosis,” “spinal-cord-injury,” “expression,” “activation,” and “functional recovery” were the most frequent key words. Additionally, “transplantation,” “mesenchymal stemness-cells,” “therapies,” “activation,” “regeneration,” “repair,” “autophagy,” “exosomes,” “nlrp3 inflammasome,” “neuroinflammation,” and “knockdown” were the latest emerging key words, which may inform the hottest themes.

**Conclusions:**

Apoptosis after SCI may cause the ultimate neurological damages. Development of novel treatments for secondary SCI mainly depends on a better understanding of apoptosis-related mechanisms in molecular and cellular levels. Such therapeutic interventions involve the application of anti-apoptotic agents, free radical scavengers, as well as anti-inflammatory drugs, which can be targeted to inhibit core events in cellular and molecular injury cascades pathway.

## 1 Introduction

Apoptosis, also known as programmed cell death, plays a critical role in spinal cord injury (SCI). Apoptosis is a kind of active cell death featured by internal cell controls and unique cell morphology (Raff et al., 1993; Nagata, 1997). Apoptosis can be contrasted to necrosis, where cell membranes are compromised, cells swell and lyse, and cell death is a passive response to destructive injuries out of cell controls (PorteraCailliau et al., 1997). Apoptosis can be activated by various mechanisms, which includes free radical damage, glutamatergic excitotoxicity, inflammatory injury, and cytokines release. Apoptosis also plays a significant part in modulating the type and proportion of immunocytes and in suppressing uncontrolled cellular proliferation which may lead to tumor (Steller, 1995); it might also be critical in eliminating cells via irreparable DNA damage (Roos and Kaina, 2013). Spinal cord injury (SCI) is a common type of neurological trauma which results in sensory and motor deficits below the lesion sits. Severe SCI may even lead to death or emotional, physical, and economic consequences for patients, the families, and society (Badhiwala et al., 2019). SCI can be non-traumatic or traumatic. Traumatic SCI is caused by direct mechanical damages including compression and contusion and, whereas impaired circulation and infection are the etiologies of non-traumatic SCI (Walker et al., 2001; Alito et al., 2021). The local ischemia, edema, and hypoxia induced by the injuries within the spinal cord and surrounding tissues, are the major causes of neuronal apoptosis and irreversible spinal cord dysfunction (Anjum et al., 2020). Therefore, it is crucial to hinder the progression of SCI to neuronal apoptosis. Currently, the functional significance of apoptosis and its mechanisms in SCI are under intense investigation.

The field of apoptosis and SCI has produced a large volume of important clinical and scientific studies over the years, which could be found in prestigious journals with high impact factor. These studies are spread over various different journals, which makes it difficult to determine which of them has been the most influential in apoptosis and SCI. Currently, no study has performed bibliometric approaches to realize in-depth mining and investigate the research field of apoptosis and SCI. Therefore, it is of vital significance to perform bibliometric analysis to investigate the related studies, which can provide an overview and identify promising future directions.

Web of Science (WOS) is a crucial scientific citation index tool, which is one of the most authoritative database for technical and scientific literature (Li et al., 2018). Science citation index (SCI) citation search system of WOS could help users to analyze the value of researches from the perspective of literature citation and construct a reference co-citation network of unique topics. Bibliometrics can quantitatively investigate all knowledge carriers through statistical approaches. It is a comprehensive knowledge system, which combines bibliography, statistics, and mathematics. It is a powerful tool for analyzing possible trends in scientific archives and identifying critical hotspots via exploring the characteristics of literatures and databases (Huang et al., 2023b). Bibliometric analysis has been utilized in various medical fields, such as trauma, rheumatism, and endocrinology (Huang et al., 2023a,b; Zhu et al., 2023). However, it remains a lack of bibliometric view on the field of apoptosis and SCI. Hence, by using a comprehensive and systematic bibliometric analysis, this study analyzed the articles regarding apoptosis and SCI to investigate the current states and estimate research hot topics and development trends within this field based on WOS.

## 2 Materials and methods

### 2.1 Data source and retrieval strategy

The Web of Science™ (Clarivate™, Philadelphia, PA, USA) was used for publication retrieval on 1st Oct 2023. The retrieval strategy was as follows: (TS = “spinal cord injury” OR TS = “spinal injuries” OR TS = “spinal cord injuries” OR TS = “spinal injury” OR TS = “spinal cord trauma” OR TS = “spinal cord laceration” OR TS = “post-traumatic myelopathy” OR TS = “spinal cord contusion” OR TS = “spinal cord transection”) AND (TS = “apoptosis”). Articles and reviews in English were selected across multiple different publications. A total of 3,425 documents published from 1st January 1994 to 1st Oct 2023 were retrieved from the WOS Core Collection (WOSCC). After excluding documents which did not meet the requirements of article type and language, the remaining documents were further evaluated based on the title and abstract. The raw data could be seen in Supplementary File 1. For avoiding bias caused by the frequent database update, literature retrieval and extraction of data were conducted on 1st Oct, and the processed data were imported into Bibliometric tools for the subsequent investigation.

### 2.2 Data analysis

All data were transformed into a text format, which were investigated by utilizing Bibliometrix package (version 3.2.1) in R (version 4.2.0, Institute for Statistics and Mathematics, Vienna, Austria^1^) to conduct comprehensive visualization, as well as knowledge mapping analysis (Aria and Cuccurullo, 2017). Biblioshiny software was applied for analyzing and visualizing the processed data. Visualization of annual scientific production and average citations indicated development trends within the field of apoptosis and SCI. The impact of countries, institutions, journals, and authors was investigated based on the visualization of multiple bibliometric indicators, including annual publications, local/global citations, and H-index. Specifically, H-index is frequently used to assess scholars’ scientific outputs as well as influence. Inter-country and/or inter-author cooperative relations were also evaluated (Glanzel, 2008). Country cooperative networks and author cooperative networks were also constructed to show the cooperative relationship among them. Subsequently, highly cited literatures and high-frequency key words were determined. A key word co-occurrence network together with a historical direct citation network map was constructed to expound on the research hot topics in the field of apoptosis and SCI. Herein, countries, affiliations, authors, co-cited documents, and frequent key words associated with the field of apoptosis and SCI were investigated and visualized. The dimensionality reduction technique was also utilized for analyzing the conceptual structure of key words, which can help identify the development trends and critical hot topics of apoptosis and SCI.

## 3 Results

### 3.1 Analysis of annual publications

**FIGURE 1:**
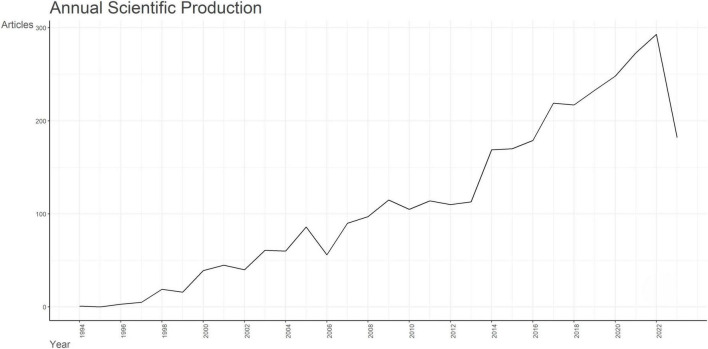
Analysis of annual scientific production of documents on apoptosis and spinal cord injury.

Data retrieval strategies and collection processes for this study were shown in Supplementary Figure 1. From 1st January, 1945 to 1st October, 2023, 3,425 English-language articles were retrieved. A total of 3,359 articles that satisfied our criteria including 2,922 (87.0%) research articles and 437 (13.0%) review articles were extracted for further analysis. From 1994 to 2008, few literatures were published in the field of apoptosis and SCI, with annual publications less than 100. Since the year 2013, the annual publications have grown fastest. From 2013 to 2014, the number of associated articles showed its largest increase (more than 100 articles), which indicated that, from 2013, increasing numbers of researchers began to concentrate on the field of apoptosis and SCI. The data retrieval date (1st October 2023) may account for the steep decline in 2023 (Figure 1). In brief, it indicated the field of apoptosis and SCI is becoming more and more attractive, which shows great potential for in-depth exploration.

### 3.2 Analysis of countries, affiliations, and authors

**FIGURE 2:**
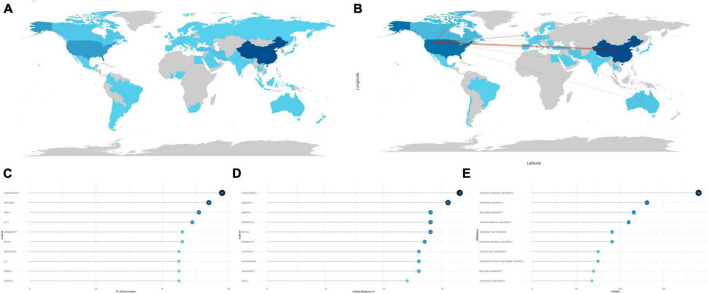
Analysis of countries, authors, and institutions. **(A)** Country scientific production map. The darker the color, the greater the number of documents (frequency) published by a country or region. **(B)** Country collaboration map. Red lines represent the collaboration bonds between countries. The thicker the line, the stronger the collaboration between countries. **(C)** Most relevant authors in the field of apoptosis and spinal cord injury. The size and darkness of the nodes are in proportion to the number of documents published by a particular author. **(D)** Most influential authors, as measured by the h-index. The size and darkness of the nodes are in proportion to the h-index for each author. **(E)** Most relevant affiliations in the field of apoptosis and spinal cord injury. The size and darkness of the nodes are in proportion to the number of documents produced by the affiliation.

Ranked by utilizing accumulated publication frequency, the top 5 most productive countries participating in the field of apoptosis and spinal cord injury were the China (1,738 publications), the USA (611 publications), Japan (128 publications), Italy (105 publications), and Korea (99 publications) (Figure 2A). In addition, the USA (43,453 citations), China (30,504 citations), Canada (7,476 citations), Japan (5,301 citations), and Italy (4,186 citations) were the five most cited countries, which indicated excellence in quality and quantity of the researches (Supplementary Figure 2A). The USA showed strong collaboration bonds with China, Canada, the UK, and Korea (Figure 2B). Collaboration strength across different countries could be reflected by the single-country publication (SCP) and multiple-country publication (MCP) rates. Further, countries exhibiting the highest MCP ratio included the USA, China, Iran, Italy, and Canada; other countries, including Japan, Turkey, and Korea, concentrated more on the articles published domestically (Supplementary Figure 2B).

During the last decades, many scholars have made tremendous efforts to promote the development of this research field. Lotka’s law showed interactions between different authors and their publications (Coile, 1977). Generally, it showed a small proportion of authors produced the main publications in this field. Here, the Lotka’s law was utilized for evaluating the publications in apoptosis and SCI, as the vast majority of the related articles in apoptosis and SCI were produced by a small part of authors (Supplementary Figure 2C). Besides, the top 10 most prolific authors were illustrated by Figure 2C, and Cuzzocrea S, Mazzon E, Xiao J, Liu J, and Bramanti P accounted for the top 5. Based on the H-index, the top 10 most influential authors were shown in Figure 2D, with the H-index greater than 20, indicating that they produced 20 documents, all with a citation number above 20. Further, Cuzzocrea S and Mazzon E were also the two most influential authors in the field of apoptosis and SCI. Nevertheless, publication number of Cuzzocrea peaked in 2017, while he gradually tapered off production of the articles after 2017, whereas Xiao J and Mei XF, although starting late, have retained active roles until today. Liu J, Li Y, Wang J, and Zhang Y continued to be active after 2017 (Supplementary Figure 2D).

It is important to identify the prolific and influential institutions in the field of apoptosis and SCI. Figure 2E indicated that Wenzhou Medical University ranked first, with 190 publications, followed by Nantong University (131 publications), Zhejiang University (116 publications), Jinzhou Medical University (110 publications), and University of Toronto (91 publications). These findings were in good agreement with results discussed above, which showed China and Canada were the pioneers of this field.

### 3.3 Analysis of sources

**FIGURE 3:**
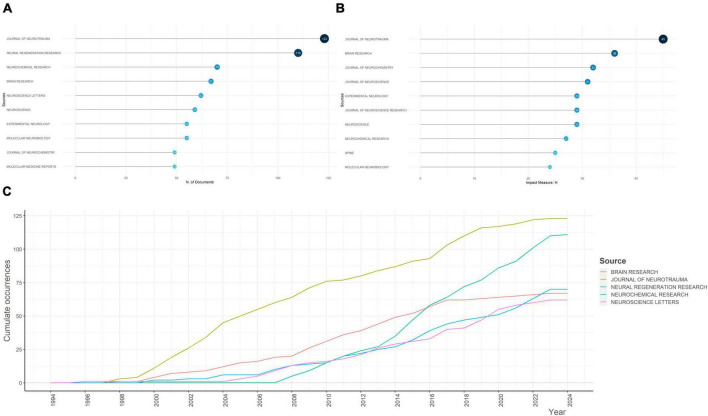
Analysis of the most relevant sources for apoptosis and spinal cord injury and their growth. **(A)** The top 10 most relevant sources for apoptosis and spinal cord injury. The diameter and darkness of the node are in proportion to the number of documents published by the source. **(B)** The top 10 sources with the highest h-index. The diameter and darkness of the node are in proportion to the h-index of the source. **(C)** Sources’ growth from 1945 to 2023.

Since 1994, a variety of documents have been published from 770 sources, and the top 10 most popular sources were identified (Figure 3A). *Journal of Neurotrauma* (123 publications) and *Neural Regeneration Research* (110 publications) were the top two, with number of publications over 100, which accounted for the majority of associated publications (Supplementary Figure 3). Further, taking impact of journals into consideration, *Journal of Neurotrauma* ranked the first (H-index = 45), followed by and *Brain Research* (H-index = 36), *Journal of Neurochemistry* (H-index = 32), and *Journal of Neuroscience* (H-index = 31) (Figure 3B). During the last decades, cumulated co-occurrences of mainstream sources presented a significant increase after 1997, indicating an upsurge in popularity and significance of the aforementioned journals. *Neural Regeneration Research* did not publish papers until 2007, but this journal climbed to the second place in 2023. The detailed trends were presented in Figure 3C.

### 3.4 Analysis of cited documents and references

**FIGURE 4:**
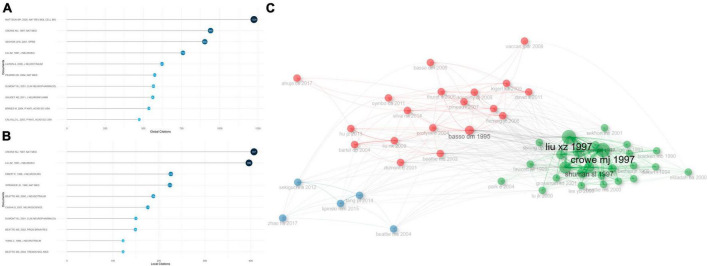
Analysis of the most cited documents in apoptosis and spinal cord injury, and their correlations. **(A)** The top 10 most globally cited documents concerning apoptosis and spinal cord injury. The size and darkness of the nodes are in proportion to the number of global citations of each document. **(B)** The top 10 most locally cited documents concerning apoptosis and spinal cord injury. The size and darkness of the nodes are in proportion to the number of local citations of each document. **(C)** Historical direct citation network. Each dot represents a document and is labeled with first author’s surname and the publication year.

Most cited documents and references played as future directions for the investigation into apoptosis and SCI. Globally cited documents were the articles that were cited in the WOS, while locally cited documents were those that were cited in apoptosis and SCI-related documents. Therefore, high publication numbers of globally cited documents reflected the general influence, while high publication numbers of locally cited documents suggested their impacts in the field. Figures 4A, B showed the top 10 globally cited documents as well as the top 10 locally cited documents. Specifically, the highest globally cited literature, “Apoptosis in neurodegenerative disorders,” by Mattson (2000) (1,224 global citations), summarized the significant genetic and environmental factors responsible for human neurodegenerative disorders including SCI, which provided evidence for a common pathway of neuron death - apoptosis - including perturbed calcium homeostasis, oxidative stress, caspases activation, and mitochondrial dysfunction. Neuronal apoptosis is counteracted by survival signals that can stabilize mitochondrial function and inhibit oxyradicals. Importantly, with the investigation of mechanisms that either prevent or promote cell apoptosis come novel treatment strategies for SCI.

The second highest globally cited and the highest locally cited document, “Apoptosis and delayed degeneration after spinal cord injury in rats and monkeys,” by Crowe et al. (1997) (940 global citations, 407 local citations), identified apoptotic cells from 6 h to 21 days post SCI in rats, especially in white matter of spinal cord, and the apoptotic cells were mostly positive for oligodendrocyte biomarkers. Furthermore, following SCI in monkey models, the apoptotic cells were observed in remote fiber tracts showing progressive degeneration. Both secondary degenerating events within the lesion sites of SCI and chronic demyelination of spinal tracts that were away from the sites appeared to be associated with apoptosis. The third highest globally cited document, “Epidemiology, demographics, and pathophysiology of acute spinal cord injury,” by Sekhon and Fehlings (2001) (902 global citations), summarized the mediators of secondary injuries post SCI, including vascular dysfunction, excitatory amino acids, free radicals, sodium, calcium, inflammatory responses, and apoptosis. The second highest locally cited document, “Neuronal and glial apoptosis after traumatic spinal cord injury,” by Liu et al. (1997) (396 local citations), showed apoptosis dependent on active protein synthesis may result in glial and neuronal cell-death, and cause neurological dysfunction in traumatic SCI rats.

Co-citation historical graph of the most cited documents (more than 50 times) was illustrated in Figure 4C. Documents that appeared closer in this figure shared stronger relationships. The most cited documents were classified into three sub-clusters. In great agreement with the co-citation relationships, the articles published by Crowe et al. (1997) distributed in the vicinity of Liu et al. (1997), indicating their strong correlations.

The aforementioned articles were pioneering articles in the field of apoptosis and SCI. These articles exhibited a high inheritance level, which indicated that new findings were derived from the preliminary foundation. The dominant position of these documents could be identified in the bar plot and Sankey diagram (Supplementary Figures 4A, B).

Among the references that were most locally cited (Supplementary Figure 4A), “A sensitive and reliable locomotor rating-scale for open-field testing in rats” (Basso et al., 1995), by Perry, published in 1995 (571 local citations), developed an expanded, efficient, and unambiguous locomotor rating scale for standardizing locomotor outcome measures among various laboratories, which can clearly distinguish behavioral outcomes after different injuries and predict anatomical alterations in the injured sites. “Apoptosis and delayed degeneration after spinal cord injury in rats and monkeys” (Crowe MJ, 1997, 407 local citations) (Crowe et al., 1997) ranked the second in the most locally cited references, followed by “Neuronal and glial apoptosis after traumatic spinal cord injury” (Liu XZ, 1997, 396 local citations) (Liu et al., 1997).

### 3.5 Identification of frontiers and hotspots

**FIGURE 5:**
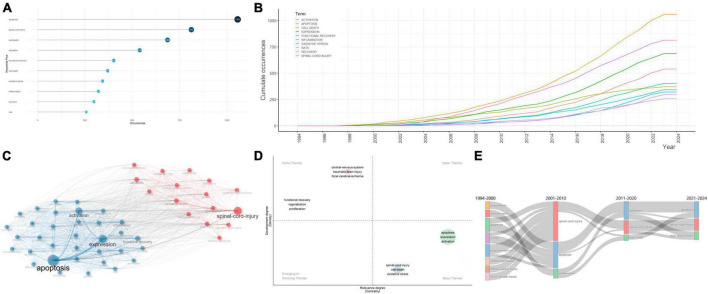
Analysis of keywords in articles regarding apoptosis and spinal cord injury. **(A)** The top 10 most frequent key words selected from KeyWords Plus. The size and darkness of the nodes are in proportion to the number of occurrences of each keyword. **(B)** Evolution, from 1945 to 2023, of the keywords. **(C)** Co-occurrence analysis of key words. Keywords were divided into two clusters labeled with different colors (blue, red). **(D)** Thematic map of themes. The key themes were each labeled with core keywords chosen to be representative of the theme. **(E)** Sankey plot of the most frequent key words in different time period.

Key words constantly appear in internalized documents reflect the hot topics in a unique field. As a useful algorithm that is unique to Clarivate databases, KeyWords Plus promotes the power of cited-reference searching by searching across disciplines for all the documents that had cited references in common. Because KeyWords Plus was generated from cited titles, the key words cannot be changed. Based on KeyWords Plus analysis (Figure 5A), we identified “apoptosis” (1,057 occurrences), “spinal-cord-injury” (812 occurrences), “expression” (686 occurrences), “activation” (539 occurrences), and “functional recovery” (402 occurrences) were the top 5 most common key words among the studies, far exceeding all other key words, indicating that the field focused on apoptosis-related gene expression and activation, as well as functional recovery after SCI. The high frequency of appearance of “apoptosis,” “spinal-cord-injury,” “expression,” “activation,” and “functional recovery” was illustrated by the keyword tree and word cloud (Supplementary Figures 5A, B).

It is important to explore the evolution trends of key words in the field of apoptosis and SCI. During the time period from 1994 to 2023, occurrence of all key words showed a general uptrend. Among all keywords, “apoptosis,” “spinal cord injury,” “expression,” and “activation” showed robust growth, which exhibited promising future development (Figure 5B). To predict future hotspots of the field, several key words showing high frequency in the past three decades were revealed.

Specifically, the minimum frequency of key words was set to 10, and the number of key words each year was set up to 3. Specifically, 66 key words were involved, and their frequency and popular periods were investigated (Supplementary Figure 6A).

Key words between 2000 and 2005 were of relatively low frequency and density, concentrating on “brain injury,” “DNA fragmentation,” “programmed cell-death,” “spinal-cord-injury,” “cerebellar granule cells,” “transgenic mice,” “caspase-3 apoptotic cascade,” “interleukin-1-beta converting-enzyme,” “compression trauma,” “controlled trial,” “blood-flow,” and “glial apoptosis,” indicating that the morphological characteristics of apoptotic cells in animal models, as well as clinical trials have consistently been the research centers in the field of apoptosis and SCI. Previous studies have also shown potential benefits of peptides that inhibit the activity of interleukin-1-beta converting-enzyme-like proteases (caspases) using ischemia models (Takagi et al., 2003). Caspases were well accepted to be critical parts in the effector system of cell apoptosis (Pan et al., 2018).

From 2006 to 2010, both the abundance and frequency of new key words increased, which suggested the research topics became more concentrated within the period. The typical key words included “polymerase,” “experimental allergic encephalomyelitis,” “cytochrome-c,” “peroxynitrite,” “amyotrophic-lateral-sclerosis,” “tumor-necrosis-factor,” and “messenger-rna,” suggesting the studies concentrated on the apoptosis-related molecular alterations, cytokines release, and Inflammation in animal experiments in SCI. *In vitro* experiments indicated that, cortical neurons that were exposed to stretch injury showed increased vulnerability to apoptosis-related DNA fragmentation, cytoplasmic cytochrome-c accumulation, and peroxynitrite formation (Lau et al., 2006).

Key words between 2011 and 2015 showed high frequency and density, focusing on “nitric-oxide synthase,” “cerebral-ischemia,” “*in vivo*,” “adult rats,” “degeneration,” “neuronal apoptosis,” “*in vitro*,” and “cell death,” which suggested that studies in this period focused on the free radicals and cell damages in SCI based on *in vivo* and/or *in vitro* animal experiments. These key words then continued into 2020 and may even become hotspots in the future. This phenomenon could be partially ascribed to the quick development of investigation in apoptosis and SCI. A unique free radical in neurons that has attracted highly attention is nitric oxide (NO), which is generated by nitric oxide synthase (NOS). Excessive synthesis of NO may result in generation of the NO radical NO2, which may lead to the injury of neuronal cells and cause neuronal degeneration in multiple approaches, such as combining with O_2_- to generate the highly reactive peroxynitrite radical ONOO^–^ (Jiao et al., 2010). Moreover, the effects of NOS inactivation in SCI and cerebral-ischemia were complicated by the conflicting neurotoxic activities of excessive NO in neuronal cells and the neuroprotective activities of NO on improved blood flow perfusion (Alvarado-Sanchez et al., 2019).

In the last 8 years, the key words “spinal-cord-injury,” “functional recovery,” “expression,” “apoptosis,” “autophagy,” “transplantation,” “mesenchymal stemness-cells,” “repair,” “cells,” “activation,” “regeneration,” “inflammation,” “exosomes,” “nlrp3 inflammasome,” “neuroinflammation,” “therapies,” and “knockdown” arose in the data source and lasted for approximately 3 years, which coincided with the peak in annual publications production. Therefore, we speculated that applying mesenchymal stemness cell (MSC) transplantation, inhibiting the nlrp3 inflammasome, reducing neuroinflammation, knockdown of inflammatory-activation-related factors and the inflammatory pathways in SCI might be trending topics in the future (Sun et al., 2023). Specifically, MSCs were first identified by Friedenstein et al. (1968) as a unique fibroblast-like population of adherent cells in the bone marrow. MSCs were defined by the self-renewal ability, capacity to differentiate to three distinct cell subpopulations *in vitro* (chondrocytes, osteoblasts, and adipocytes) and by their specific expression of cell surface markers (CD105, CD73, and CD90) (Pittenger et al., 1999). MSCs could be isolated from various tissues, which exhibit unique beneficial immunological properties and are relatively easier to expand *in vitro* (Pittenger et al., 2019). Further, the MSC secretomes showed paracrine effects on the local micro-environment post injury, which made them well suited for SCI therapy.

Treatments should be aimed at inhibiting inflammation-induced apoptosis, at targeting the crosstalk between autophagy and apoptosis, at inhibiting intercellular co-factors of apoptosis (e.g., exomes), or at promoting the cellular endogenous mechanisms that may suppress apoptosis during the processes of secondary SCI. Therapeutic strategies that integrate control of apoptosis with promotion of recovery mechanisms such as MSC transplantation may be potentially effective in facilitating spinal cord function recovery post SCI (Deng et al., 2021).

### 3.6 The research status of hot topics in apoptosis and SCI

Besides investigating the most frequent key words, it is of vital significance to construct a key words co-occurrence network to show the knowledge structure in apoptosis and SCI. Key words that occurred more than ten times were classified in two distinct sub-clusters, indicating two research themes or topics, respectively (Figure 5C; Supplementary Figure 6B). Cluster 1 (red), where “spinal-cord-injury,” “oxidative stress,” “cell-death,” “central-nervous-system,” “*in vitro*,” “traumatic brain-injury,” “nf-kappa-b,” “focal cerebral-ischemia,” “neuronal apoptosis,” “nitric-oxide,” “up-regulation,” “*in vivo*,” “neuronal apoptosis,” “induced apoptosis,” and “messenger-rna” were the largest nodes. It chiefly explained the critical role of free radicals and inflammation damages in SCI and focal cerebral-ischemia based on *in vivo* and/or *in vitro* experiments. Cluster 2 (blue) was represented by “apoptosis,” “expression,” “activation,” “functional recovery,” “inflammation,” “stem-cells,” “microglia,” “survival,” “transplantation,” “differentiation,” “regeneration,” “rats,” “model,” “methylprednisolone,” “neuroprotection,” and “growth,” which were probably about the mechanisms of apoptotic cells in SCI pathophysiological processes and application of stem-cell transplantation therapy and therapeutic agents. Understanding the molecular basis of apoptosis in SCI might promote glial and neuronal survival via the administration of anti-apoptotic co-factors, which can facilitate the functional recovery after SCI.

A thematic map was generated to show the density and centrality of apoptosis and SCI-related themes (Figure 5D). Density represented the development degree of specific themes, and higher density degree reflected higher maturity of the themes. Further, centrality represented the intimacy degree with various themes, and high centrality reflected the core theme. Four clusters of themes were shown on the four-quadrant graph with each quadrant indicating different research states of themes. “Central-nervous-system,” “traumatic brain-injury,” “focal cerebral-ischemia,” “functional recovery,” “regeneration,” and “proliferation” laid in the niche themes quadrant, which were highly developed, despite their relatively weak correlations with the field of apoptosis and SCI. The themes “apoptosis,” “expression,” “activation,” “spinal-cord-injury,” “cell-death,” and “oxidative stress” had solid associations with this filed while lacked development, which served as the basic themes.

In order to investigate these key words attracting varying degrees of attention in different periods, which may be associated with certain hot topics in different development stages, we constructed a Sankey plot to show the frequency of occurrences in each period (Figure 5E). Before 2000, key words mainly consisted of “apoptosis,” “model,” as well as “programmed cell-death,” suggesting that the researches concentrated on the apoptotic alterations in SCI animal models. From 2001 to 2010, more attention was paid to “proliferation,” “apoptosis,” and “spinal-cord-injury,” which remained hot topics until 2020. Since 2021, “apoptosis,” “spinal-cord-injury,” and “axon regeneration” have received continuous interest, which indicated enhancing neuroprotection effects and promoting axon regeneration after SCI became hot topics in the field.

## 4 Discussion

### 4.1 General information

Apoptosis plays a crucial role in those secondary SCI mechanisms, which may lead to the ultimate neurologic insults. A bibliometric analysis was urgently needed to comprehensively evaluate the related researches to get an overview and determine promising research directions. Here, we investigated 3,359 documents published from 1994 to 2023 regarding apoptosis and SCI.

Annual publications may represent the degree of researchers’ interest in a research field, to a certain extent. The number of researches on apoptosis and SCI began to increase dramatically following 2013, which has continued to show a general increase. Further, from 2013 to 2014, the publication number exhibited its largest increase (more than 100 articles), mainly focusing on neuronal apoptosis and degeneration. The same topics lasted for several years, till the developments of stem-cell transplantation therapy and anti-apoptotic therapeutic agents were used extensively, which gradually became the research hotspots in apoptosis and SCI. The field of apoptosis and SCI regained its popularity after 2018, and the annual publications increased up to approximately 100.

Among all the relevant countries, China ranked the first in aspect of the overall number of publications, while the USA was the most influential country. China was the home to the vast majority of most relative affiliations, including the Wenzhou Medical University, Nantong University, Zhejiang University, and Jinzhou Medical University. The USA came in second in total number of publications, exhibiting relatively high number of SCPs and MCPs. Various countries also devoted joint efforts to overcome the obstacles, and the USA had the strongest correlations with China, Canada, the UK, and Korea. The USA exhibited the highest MCP ratio, suggesting that most of the articles published by the USA were published through collaborating with other countries. As for the most relevant authors, Cuzzocrea S, Mazzon E, Xiao J, Liu J, and Bramanti P accounted for the top 5 most productive authors, and most of them are still active today. Several connections could be observed across the most relative authors and affiliations. Wenzhou Medical University, ranking the first across the most relevant affiliations, was the institution where Xiao J, the most productive Chinese author in this field, worked. It showed the most relative authors played significant parts in the development of exceptional platforms.

*Journal of Neurotrauma* and *Neural Regeneration Research* were the top two most relevant sources, while *Journal of Neurotrauma*, *Brain Research*, and *Journal of Neurochemistry* were the three most influential sources, which were valuable sources for apoptosis and SCI-related researches.

Most locally cited or globally cited documents functioned as the milestones and crucial stimuli for future researches. “Apoptosis and delayed degeneration after spinal cord injury in rats and monkeys,” by Crowe et al. (1997), demonstrated in monkeys that apoptosis may lead to the secondary degeneration in lesion sites of SCI and chronic demyelination of tracts away from the injured sites. Apoptotic cells were located along the long descending and ascending tracts, which were adjacent to myelin sheaths within the white matter, and many of these cells were oligodendrocytes. This article also motivated the study “Spinal cord injury: molecular mechanisms and therapeutic interventions” (Hu et al., 2023), by Cheng LM, published in 2023, which summarized the latest progress associated with neuronal regeneration as well as circuit reconstruction, proposing potential research directions for SCI recovery and rehabilitation.

Importantly, with the development of single-cell RNA sequencing (scRNA-seq) and spatial transcriptome technology, as well as bio-active materials and stem-cell transplantation, more and more researches concentrated on constructing intermediate neuronal networks to enhance neuronal regeneration and neuronal circuit reconstruction, rather than on enhancing regeneration of axons along the long descending and ascending tracts. Another perspective on the scRNA-seq technology, temporal molecular, and apoptosis-related cellular alterations after SCI was provided by the article “Temporal and spatial cellular and molecular pathological alterations with single-cell resolution in the adult spinal cord after injury” (Li C. et al., 2022), by Cheng LM, published in 2022, which identified new astrocyte and microglia subtypes within the uninjured spinal cord, as well as their dynamic conversions into unique stage-specific subtypes. Microglial activation accompanied with apoptosis in neurons occurred at 3 days after SCI and by 14 days post SCI, indicative of the second attack waves. Altogether, it could be concluded that the new technique has been applied widely which has extremely accelerated and promoted the process of the field of apoptosis and SCI.

### 4.2 Hotspots and frontiers

**FIGURE 6:**
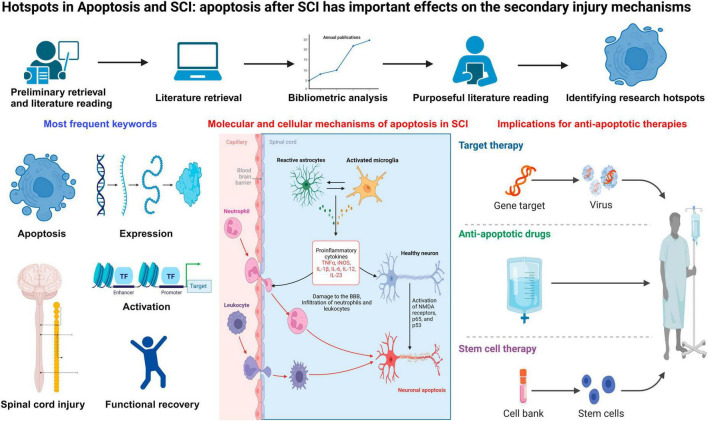
The hotspots and development frontiers in apoptosis and spinal cord injury.

The most frequent key words reflected the core research themes of this field. “apoptosis,” “spinal-cord-injury,” “expression,” “activation,” and “functional recovery” were the most frequent key words identified in the course of studying apoptosis and SCI. Additionally, “transplantation,” “mesenchymal stemness-cells,” “therapies,” “activation,” “regeneration,” “repair,” “autophagy,” “exomes,” “nlrp3 inflammasome,” “neuroinflammation,” and “knockdown” were the latest emerging key words in recent years. By visualizing the density and overlay of key words and the most significant citation bursts, we identified the hot topics in apoptosis and SCI as described in the following sections (Figure 6).

### 4.3 Evidence supporting the observation of apoptosis after SCI

Apoptosis has been identified in animal SCI models and human traumatic and ischemic SCI. Specially, apoptosis occurred in oligodendroglia, astrocytes, microglia, as well as neurons in spinal cord of rats post severe damages (Yong et al., 1998). Most of the apoptotic oligodendrocytes were identified at the longitudinal tracts of white matter (Li et al., 1996). Apoptotic neuronal cells were observed at 4 h which peaked at 8 h post SCI; apoptotic glial cells were identified at 4 h which reached to the highest level at 24 h post SCI; apoptotic oligodendrocytes were identified at 24 h which peaked at 8 days in the white matter post SCI (Liu et al., 1997; Shuman et al., 1997). Apoptotic microglia were relatively rare at 24 h, but gradually increased and peaked at 8 days post SCI (Shuman et al., 1997). Further, apoptotic cells can mainly be identified in the rim of surviving tissues near the center in the damaged spinal cord, which might partially explain why the injured site progressively expand (Lu et al., 2000). Moreover, apoptosis could be observed in the oligodendrocytes in white matter tracts exhibiting Wallerian degeneration, and it is an important feature of the chronic SCI (Emery et al., 1998; Lu et al., 2000).

### 4.4 Alterations of the apoptosis-related mechanisms in SCI

Apoptosis is the most frequent form of programmed cell-death, which is well accepted as a physiological process occurring naturally, playing an important role in secondary SCI. Free radicals, excitotoxins, and inflammation regulators are the factors which can trigger cell-death and activate apoptosis (Lu et al., 2000). Glutamate damages neuronal cells via allowing Ca^2+^ entry, either as a result of depolarization leading to activation of voltage-gated Ca^2+^ channels or via opening ligand-gated channels, a-amino-3-hydroxy-5-methyl-4-isoxazolepropionic acid (AMPA), and N-methyl-D-aspartate (NMDA) receptors. Overactivation of the NMDA receptor due to excessive glutamate can initiate a cascade of molecular and cellular events including excess Ca^2+^ entry, mitochondrial dysfunction, followed by cell apoptosis. After SCI, the secondary attack waves are suggested to be resulted from the continuation of cell destruction caused by apoptosis, while the long-term neurological damages may be due to a wide range of neuronal apoptosis as well as apoptosis of oligodendrocytes within the lesions.

Apoptosis is initiated via the mitochondrial (intrinsic) pathways and death receptor regulated (extrinsic) pathways (Iorga et al., 2017; Sharma and Kanneganti, 2017; Thornton et al., 2017). Extrinsic pathways can initiate the activity of death receptors, such as tumor necrosis factor-α (TNF-α) and Fas, which eventually results in caspase-8 activation (Elmore, 2007). Upon binding to the receptors, Fas can activate Fas-associated death domain (FADD) and promote the binding of ligand TNF-α to its receptor (TNF-α receptor, TNFR), which can activate TNFR1-associated death domain protein (TRADD) and facilitate the recruitment of FADD (Reed, 2000). Subsequently, death-inducing signaling complex (DISC) is formed to activate procaspase-8. Additionally, intrinsic apoptotic pathways directly act without receptor mediators, which can lead to the release of mitochondrial cytochrome C (cyto-C) and activation of caspase-3 and caspase-9 (Elmore, 2007). In parallel, intrinsic apoptotic pathways enhance fragmentation of DNA, resulting in apoptosis. Bcl-2 expression is triggered, which can alter the release of cyto-C in these pathways. Besides, Bcl-2 is accumulated within the outer membrane, which modulates intrinsic cascades pathways to induce apoptosis. Members of Bcl family consist of various pro-apoptotic proteins, as well as multiple anti-apoptotic proteins (Leibowitz and Yu, 2010). Pro-apoptotic regulatory proteins may enhance the permeability of mitochondrial membrane, and facilitate the release of cyto-C to cytosol. Cyto-C can bind apoptotic protease activating factor-1 (Apaf-1) and caspase-9 to form the apoptosome complex, which in turn leads to the activation of caspase-9 as well as other caspases (Gogvadze et al., 2006).

Reactive oxygen species (ROS) may activate poly (ADP-ribose) polymerase-1 (PARP-1). Besides, PARP-1 substrate for caspases can decrease ATP and β-nicotinamide adenine dinucleotide (NAD), which can promote the secretion of apoptosis-inducing factor (AIF) (Sosna et al., 2014). AIF can be transferred into nucleus, which then cleaves the DNA. Finally, cell death may happen if DNA damages are not repaired (Liu and Xu, 2012). SCI generates more ROS, which leads to oxidative damages and pathological micro-environment (Visavadiya et al., 2016).

Spinal cord injury can also lead to excessive activation of PARP-1, promoting apoptosis via utilization of NAD and AIF release (Meng et al., 2015). The capability of PARP-1 cleavage in DNA repairing is weak. When extensive utilization of ATP is stagnated, enough energy is ready for subsequent apoptosis processes (Meng et al., 2015). PARP-1 degradation is promoted 6 h after SCI (Keane et al., 2001), which reaches the peak level on 3 days after SCI, and gradually disappears by 28 days post SCI (Gao R. et al., 2016).

A better understanding of the molecular and cellular mechanisms of apoptosis in SCI could help us identify potential therapeutic biomarkers. Recently, Liu et al. (2014) performed proteomics analysis to identify a core protein, endoplasmic reticulum protein 29 (ERp29), which can modulate multiple genes related to apoptosis including caspases and Erk, and promote functional recovery in rat SCI models. Moreover, minocycline, antibody blocking of the Fas ligand and blocking of inducible nitric oxide synthase (iNOS) induced by glycosphingolipid have been demonstrated to inhibit neuronal and glial apoptosis, which may improve neurological function and enhance the efficacy of stem-cell transplantation therapy (Beattie, 2004). Additionally, a previous study revealed that Caspase recruitment domain family member 6 (CARD6) played a core role in suppressing apoptosis via inhibiting release of cyto-C to cytosol and repression of caspase-3 signaling pathway (Wang J. L. et al., 2019). Zhang H. W. et al. (2020) identified that upregulation of p38 was associated with neuroinflammation and apoptosis post SCI, and p38 inhibitor SB203580 therapy may alleviate secondary SCI via inhibiting apoptosis and inflammation. The deficiency of progranulin, a 593 amino acid secreted glycoprotein, is not conducive to functional recovery after SCI through promoting cellular apoptosis and neuroinflammation (Wang C. et al., 2019). A previous analysis revealed that metformin could promote the expression of β-catenin and brain derived neurotrophic factor (BDNF), suppress apoptosis of neuronal cells and neuroinflammation, which can facilitate loco-motor functional recovery in rat SCI models (Zhang T. et al., 2020).

The secondary phase of SCI is mainly featured by loss of mitochondrial function and ATP-dependent cell dysfunction, which may trigger apoptosis. Nevertheless, the mechanisms underlying apoptosis after SCI still required in-depth investigation, and the most closely or most important genes and signaling pathways were discussed in the following.

### 4.5 Caspases in apoptosis post SCI

Apoptosis is known as a programmed cell-death modality, which is primarily modulated by the caspase family of cysteine proteases (Green, 1998). Previous studies have demonstrated that 6 h after injury, caspase-8 and 9 were identified on injured lesions of the gray matter in spinal cord (Eldadah and Faden, 2000; Keane et al., 2001). Almost 3 days after injury, caspase-9 and 8 were also identified in the gray matter and by the 7 days after injury, and these caspases were observed in both gray and white matters. Approximately 1-day post SCI, increase of caspases was firstly identified in the gray matter and subsequently in the white matter (Keane et al., 2001).

Downstream or upstream factors of caspase-3 apoptotic pathway have been demonstrated to be upregulated in rats post SCI, playing an important part in apoptosis (Springer et al., 1999). Caspase-3 plays a critical part in the white and gray matter of spinal cord post injury (Chen et al., 2011). A previous study identified the activation of caspase-3 in both neuronal cells and oligodendrocytes on 7 days post injury (Takagi et al., 2003). Activated caspase-3 appears in the oligodendroglia within the lesions of spinal white matter at early stage after SCI. Besides, activation of caspase-3 was also identified in some dorsal, whereas not ventral, horn neuronal cells post SCI (McEwen and Springer, 2005).

### 4.6 FasL in apoptosis post SCI

Fas/FasL signaling pathways play critical parts in activating caspase-8 and extrinsic apoptosis pathways. Fas molecule is composed of three transmembrane, cytoplasmic, and extracellular domains. Fas and Fas ligand (FasL) was demonstrated to activate apoptosis-initiating proteases and apoptosis (Lagunas-Rangel, 2023). Fas and FasL permit the activation of caspase-8, which are implicated in the modulation of apoptosis. Expression of Fas and FasL in the lesion site is lost in 1 day after SCI, whereas increasing in tissues adjacent to the lesions (Casha et al., 2005). A previous analysis indicated the proportion of cells expressing Fas was significantly elevated in 2 h post injury (Zurita et al., 2001). Increased expression of Fas and FasL after SCI leads to extensive activation of microglial cells and pro-inflammatory pathways, which may induce apoptosis associated with neuronal degeneration, demyelination, and dysfunction (Yu and Fehlings, 2011). Fas neutralization may inhibit the infiltration degree of pro-inflammatory cells and apoptotic processes at early stage of SCI, which can reduce inflammatory responses and improve motor functional recovery post-SCI (Chen et al., 2011; Yu and Fehlings, 2011). Besides, it indicated the activation of Fas death receptor pathway played as a vital role in the apoptosis of oligodendrocytes, microglial cells, and neurons in SCI animal models (Casha et al., 2001; Zurita et al., 2001; Yoshino et al., 2004). Cross-talk between Fas and Fas receptor (FasR) could initiate apoptosis through activating the cysteine proteases, which leads to DNA cleavage and proteolysis via effector caspases that are culminated in programmed cell-death (Nagata and Golstein, 1995; Rowland et al., 2008).

### 4.7 TNF-α in apoptosis post SCI

Inflammatory responses to SCI are significant for triggering apoptosis. TNF-α is a pro-inflammatory cytokine implicated in multiple pathways to regulate apoptosis (Kim J. J. et al., 2010), which is expressed in active microglia, astroglia, oligodendroglia, and neuronal cells within the early stage of SCI (Gao et al., 2013). The extensive demyelination caused by TNF-α is related to residual axon degradation after SCI, enhanced myelin phagocytosis induced by macrophages, and the apoptosis of residual oligodendrocytes (Chen et al., 2011). In about 30 min post injury, cells with high expression of TNF-α were identified beyond the lesions of SCI, both caudal and rostral, to the lesion center and approximately 3 to 24 h later, expression of TNF-α and interleukin-6 (IL-6) was massively increased around the injured sites.

Expression of iNOS could be continuously activated by TNF-α, which is hastily overexpressed within neurons and glial cells after injury. TNF-α can regulate apoptotic processes via triggering nitric oxide (NO) through the prior stimulation of iNOS, the expression of which reached peak value in 4 h post SCI (Yune et al., 2003). Additionally, there are two significant pathways associated with TNF-α signaling, which includes nuclear factor kappa-B (NF-κB) and pro-apoptotic signaling pathways (Wang et al., 2008). TNFR1 is a specific receptor of TNF-α, and cell death induced by TNF-α is performed via TNFR1.

Activation of NF-κB promotes the expression of cellular inhibitor of apoptosis 2 (cIAP2), which suppresses apoptotic pathways via binding with TNFR-associated factor (TRAF)-2 and repressing intracellular caspases (Kim et al., 2003; Lu et al., 2013). Additionally, TNF-α may promote glutamate-induced neuron death after SCI through two supplementary mechanisms in rats: suppressing glutamate transport in astrocytes and triggering NMDA and AMPA receptors. TNF-α may facilitate the release of glutamate in astroglia, and the complex of TNF-α/TNFR1 can activate several intracellular signaling pathways that lead to the activation of glutamate exocytosis and the generation of prostaglandin E2 (Bezzi et al., 2001). TNF-α induces caspase-3 independent apoptosis, which may promote glutamate-induced cell death of neurons. Hence, regulating pro-inflammatory events and apoptotic cascades is vitally important post injury.

### 4.8 BCL2 associated X (Bax)/Bcl-2 in apoptosis post SCI

Apoptotic protein Bax and anti-apoptotic protein Bcl-2 belong to the Bcl-2 family (García-Sáez, 2012). Bax/Bcl-2 ratio represents the progression or inhibition of apoptosis within in the lesions after SCI (Adamkov, 2019). Activation of Bax is the molecular decision of apoptosis. Bcl-2 is significantly overexpressed in the development of CNS, which crucial in modulating the protection against apoptotic cell death. It suppresses various pathways of apoptosis post SCI via inhibiting production of ROS and maintaining the structure of membrane (Wang et al., 2011). Bax can promote neuronal apoptosis via changing the mitochondrial membrane permeability. Evidence showed that knockdown of Bax significantly inhibited oligodendrocyte apoptosis after SCI (Dong et al., 2003). Bcl-2 and Bax may change the cell permeability via modulating Ca2 + ion flow (Sharpe et al., 2004). Bcl-2 plays a critical part in repressing pro-apoptotic pathways and maintaining cell survival (Hardwick and Soane, 2013). Bcl-2 expression was significantly decreased in 1 day after injury, which continued till 21 days post SCI. However, Bax expression was increased at the same time points post SCI (Kotipatruni et al., 2011). Hence, it suggested Bcl-2 suppresses apoptosis via maintaining the integrity of mitochondrial membrane, preventing the oligomerization of Bax, and modulating the activation of the caspases. Further, Bcl-2 directly inhibits release of cyto-C, which can prevent the activation of Apaf-1 and caspase-9 (Edlich, 2018). Hence, Bcl-2 activation is particularly important for promoting cell survival and optimizing therapy for SCI patients.

### 4.9 Important signaling pathways in apoptosis post SCI

The PI3K/Akt and ERK/MAPK pathways play determining roles in cellular differentiation, migration, and survival (Sun et al., 2015). Akt function as the key regulator of these signaling pathways, which can regulate the expression of cyto-C and Bcl-2 (Zhou et al., 2000). The PI3K/Akt pathway regulates apoptosis through suppressing the death genes. Nevertheless, previous studies demonstrated PI3K/Akt pathway may activate brain-derived neurotrophic factor (BDNF), which can promote survival of neuronal cells in the death processes (Yoshii and Constantine-Paton, 2007; Zhang et al., 2013). Expression of PI3K, p-Akt, and Akt peaked in 1 day post SCI, which gradually decreased in time-dependent manners; whereas their absolute expression levels were significantly higher than the normal control (Zhang P. X. et al., 2015). Glycogen synthase kinase 3β (GSK-3β) is a downstream regulator of PI3K/Akt, which plays an important part in the cell death procedure. Phosphorylation of GSK-3β inhibits mPTP opening, which can result in the cytoplasmic release of cyto-C (Zhang et al., 2013). Besides, the ERK/MAPK pathway also plays a regulatory role in BDNF-induced neuroprotective capacities (Almeida et al., 2005). After injury, ERK expression is rapidly upregulated within the SCI lesions to suppress apoptosis. Then, the ERK/MAPK signaling is triggered, which may activate the genes involved in Bcl-2 family. Moreover, while mitochondrial membrane permeability is decreased, the release of cyto-C is inhibited, therefore exhibiting anti-apoptotic effects (Quan et al., 2014).

Activation of c-Jun N-terminal kinases (JNKs) is identified in oligodendrocyte apoptosis post SCI, whereas not in neuronal apoptosis (Li et al., 2007). JNKs and interacted regulators activate MAPK cascades where MAPK kinase kinase (MAPKKK) triggers MAPKK that can stimulate MAPK. Hence, MLK3/MKK7/JNK3 pathway may induce apoptosis post SCI. JNK activation phosphorylates Bcl-2, which may induce apoptosis. Besides, JNKs can trigger c-Jun to activate Fas-induced cell death (Lin et al., 2017). Additionally, JNKs have been demonstrated to promote phosphorylation of Bcl-2-associated agonist of cell death (Bad), which enhances its pro-apoptotic properties (Yu et al., 2004). Moreover, JNKs are implicated in the TNF-α-induced apoptosis by cleaving Bid and activating caspases (Deng et al., 2003).

The JAK2/STAT3 pathway is implicated in the expression of various cytokines and growth factors, apoptosis, cellular proliferation, and differentiation (Nicolas et al., 2013). JAK2/STAT3 is non-phosphorylated normally, whereas its phosphorylation will be rapidly completed in stress conditions and remained in a stable state for about 12 h. The maximum state of p-JAK2 and p-STAT3 can be identified in 12 h, which decreases after 1 day post SCI (Xia et al., 2017). JAK2/STAT3 pathway may regulate the activity of apoptosis within the early stages of SCI. Further, inflammatory regulators such as IL-6 and TNF-α are necessary for activating JAK2/STAT3 pathway, which can promote the release of various pro-inflammatory factors to trigger cascades pathways after SCI (Xia et al., 2017). Several other important signaling pathways, such as Wnt/β-catenin signaling pathway, SIRT1/AMPK signaling pathway, and E2F1/CDK1 pathway, have also been demonstrated to be implicated in modulating apoptosis in SCI (Wu et al., 2012; Gao K. et al., 2016; Zhao et al., 2017). Taken together, repressing pro-inflammatory mediators and interconnected pro-apoptotic pathways is crucial for SCI patients.

### 4.10 MicroRNAs (miRNAs) in apoptosis post SCI

Overwhelm evidence has demonstrated the critical role of miRNAs in secondary injuries post SCI (Strickland et al., 2011; Bhalala et al., 2013; Deng and Chen, 2023). Alterations of miR-338, miR-133, miR-142-3p, miR-10a, and miR-10b are closely associated with the pathogenesis of secondary SCI (Zhou et al., 2016). Exosomes from bone marrow-derived MSCs can suppress the NF-κB signaling pathway via increasing miR-23b targeting TLR4, which regulates the oxidative stress process, alleviating the pro-inflammatory responses and apoptosis post SCI and promoting functional recovery of SCI rats (Nie and Jiang, 2021). Additionally, miR-20a, miR-21, miR-223, miR-320, miR-494, miR-497, miR-124, and miR-29b have been demonstrated to participate in the apoptosis post SCI (Jee et al., 2012; He et al., 2015; Zhao et al., 2015; Bai et al., 2021; Huang et al., 2021; Ma et al., 2022; Malvandi et al., 2022). Moreover, miR-20a, miR-124, and miR-133b are implicated in enhancing angiogenesis and promoting neuronal repair post SCI (Yu et al., 2015; Theis et al., 2017; Cui et al., 2019; Wang T. Y. et al., 2019; Danilov et al., 2020). Recently, a study indicated miR-137 may suppress apoptosis and neuroinflammation post SCI through regulating MK2 (Gao et al., 2018). Further, knockdown of lncRNA XIST may have a critical effect on suppressing apoptosis of neurons following SCI via competitively binding to miR-494 and regulating PTEN/AKT/mTOR signaling pathway (Gu et al., 2017). Besides, a previous analysis revealed that PI3K/Akt/mTOR pathway was implicated in neuronal apoptosis post SCI, which may induce apoptosis via activating the mitochondrial pathways (Wang et al., 2017).

Though more and more researches devote efforts to the extrinsic or intrinsic signals which are related to apoptosis after SCI, there are problems still remained to be solved, such as the precise mechanisms leading to apoptosis of microglia, astrocytes, oligodendrocytes, and neuronal cells following SCI.

### 4.11 Microglia apoptosis post SCI

Excessive and continuous activation of microglia has been demonstrated to be detrimental to the functional recovery post SCI (Zhou et al., 2014). Microglial apoptosis was firstly identified in the dorsal horn of the spinal cord in response to peripheral nerve damages (Gehrmann and Banati, 1995), indicating microglia may respond to injuries through apoptosis, and that there might be regulation of microglial number post SCI. A previous study showed caspase-dependent microglia apoptosis after SCI (Beattie, 2004). Currently, more and more studies have concentrated on the role of long non-coding RNAs (lncRNAs) in microglia apoptosis after SCI. Knockdown of lncRNA LEF1-AS1 may attenuate microglia apoptosis and inflammatory injuries after SCI (Cui et al., 2021). LncRNA KCNQ1OT1 may promote microglia apoptosis via the regulation of miR-589-5p/NPTN axis post SCI (Chu et al., 2022). Moreover, knockdown of lncRNA XIST may mitigate microglia apoptosis via miR-27a/Smurf1 axis (Zhao et al., 2020). A previous study showed resveratrol glycosides can ameliorate oxidative stress, suppress microglia apoptosis and neuronal death after SCI, through regulating Nrf2/HO-1 signaling pathway (Li P. et al., 2022). Regulating inflammatory responses and apoptosis of microglia after SCI may inform novel therapeutic targets for SCI patients.

### 4.12 Astrocyte apoptosis post SCI

Astrocytes are significant components of spinal cord, which are sensitive to alterations in gene expression, excitations, and hypertrophy (Bradbury and Burnside, 2019). Astrogliosis is facilitated by astrocytes and appears during the chronic phase of SCI, which starts the healing process after injury (Mekhail et al., 2012). The other major constituents of the scar tissue are pericytes and the connective tissues. Normally the number of astrocytes is 10 times higher than pericytes in spinal cord parenchyma, whereas pericytes are twice the number of astrocytes in 14 days post SCI (Bradbury and Burnside, 2019). Besides, the continuous expand of lesions and cyst formation is one of the major characteristics of SCI, which indicates ongoing astrocyte apoptosis via TLR4/MyD88 signaling (Göritz et al., 2011). A previous study has also demonstrated the apoptosis-inducing effects of M1 microglia on oligodendrocytes (Kroner et al., 2014). Formation of cyst results in syringomyelia in about one-third of SCI patients, which may cause weakness, paralysis, sensation loss, and stiffness in the shoulders, back, and limbs (Fan et al., 2016). Suppressing astrocyte apoptosis may rescue the neurotrophic function of reactive astrocytes, and thus decreasing cavity areas and promoting survival of neuronal cells which otherwise execute apoptosis in secondary SCI (Kroner et al., 2014).

### 4.13 Oligodendrocyte apoptosis post SCI

Oligodendrocyte apoptosis plays an important part in determining neurological outcomes post SCI, and the main cell loss is caused by oligodendrocyte apoptosis that significantly worsens neurological outcomes (Azari et al., 2006). Oligodendrocytes appear to be particularly sensitive to injuries induced by chemical, oxidative, radical, and mechanical factors, which may cause oligodendrocyte apoptosis. Inefficient antioxidant defense ability and relatively high iron enrichment of oligodendrocytes makes them exquisitely to oxidative stress. Exposure of oligodendrocytes to hydrogen peroxide causes upregulation of NF-κB and AP-1, crucial transcription factors involved in the process of apoptosis, while iron chelator deferoxamine may confer protective effects from oligodendrocyte apoptosis (Richter-Landsberg and Vollgraf, 1998; Vollgraf et al., 1999). Toxins used to develop SCI models, including ethidium bromide and cuprizone, may damage mitochondrial respiration and cause oligodendrocyte apoptosis (Zawadzka et al., 2023). Oligodendrocyte is the cell type that is most sensitive to radical damages in SCI, which may execute radiation-induced apoptosis (Nagayama et al., 2005). TNF-α is one of the most critical triggers of oligodendrocyte apoptosis after SCI (Schaal et al., 2007). Designated death domains of TNFR1 and its co-factors have been implicated in the activation of caspases pathway and oligodendrocyte apoptosis (Hisahara et al., 1997). Interferon-gamma increases susceptibility of oligodendrocytes to TNF-α–induced apoptosis via upregulating Fas receptor (Saito et al., 2000). Release of nitric oxide and free radicals from activated microglia may cause apoptosis of oligodendrocytes (Brandys and Chadzinska, 2021). CD4 + T cells can adhere to oligodendrocytes via the Fas receptor and trigger apoptosis in oligodendrocytes (Greenhalgh et al., 2020).

### 4.14 Neuronal cell apoptosis post SCI

Extensive inflammatory responses after SCI may result in elevated release of pro-inflammatory cytokines and neuronal apoptosis. Inhibiting neuronal apoptosis is conducive to the motor functional recovery post SCI (Guha et al., 2023). Neuronal apoptosis is identified in 4 h after SCI and reaches the peak 8 h later. A recent study showed that cutting down lncRNA XIST can reduce neuronal apoptosis post SCI through competitively binding to miR-494 and modulating the PTEN/AKT/mTOR pathway (Gu et al., 2017). Wang et al. (2017) demonstrated that the AKT/mTOR/PTEN pathway is involved in neuronal apoptosis post SCI via the activation of mitochondrial system. Recently, a study identified that targeting caspase recruitment domain family member 6 (CARD6) can inhibit oxidative stress and suppress neuronal apoptosis (Wang J. L. et al., 2019). Importantly, FasL antibody blockage, minocycline, and inducible nitric oxide synthase blocking have been demonstrated to inhibit neuronal apoptosis and improve the effects of cell transplantation treatments (Beattie, 2004). Zheng et al. (2020) identified that miR-142-3p may vanquish neuronal apoptosis in SCI rats. Deficiency of progranulin may promote neuronal apoptosis and inflammatory responses, which compromises healing processes after SCI (Wang C. et al., 2019). A recent study showed that increased p38 was associated with neuronal apoptosis and neuroinflammation in SCI rats (Zhang H. W. et al., 2020). Suppressing neuronal apoptosis and neuroinflammation using SB203580, the p38 inhibitor, may be beneficial to SCI patients. Moreover, metformin may increase the expression of β-catenin and neurotrophic factors, reduce neuronal apoptosis and neuroinflammation, and improve functional recovery in SCI rats (Zhang T. et al., 2020). Conquering neuronal apoptosis after SCI has great therapeutic ramifications.

### 4.15 Fibroblast apoptosis post SCI

Fibroblasts detaching from the perivascular cells and meninges may invade and reside within lesion sites post SCI, which secrete extracellular matrix to promote fibrotic scar formation with accessory glia limitans (Zhu Y. J. et al., 2015). Migration of fibroblasts to the epicenter of injured sites leads to the fibrotic scar formation, which may impede axonal regrowth through constituting a biochemical and physical barrier (Zhu Y. et al., 2015). Therefore, modulation of apoptosis in spinal fibroblasts may suppress the formation of fibrotic scar after SCI, which can create an enabling environment for neuronal regeneration and enhance functional recovery post SCI. Mitomycin C (MMC) was demonstrated to show anti-proliferative effects via activating apoptotic pathways in fibroblasts (Liu et al., 2010). MMC may induce extrinsic and intrinsic apoptotic pathways to trigger fibroblast apoptosis (Pirnia et al., 2002). Additionally, extensive proliferation of fibroblasts is also critical in epidural scar adhesion after spinal cord decompression surgery. Promoting apoptosis in human epidural scar fibroblasts (HESFs) is an efficient way to prevent postoperative epidural scar adhesion (Sun et al., 2016). A recent study showed MMC can inhibit proliferation of HESFs and trigger apoptosis in these cells following surgical decompression for SCI, partially through the endoplasmic reticulum stress pathway (Sui et al., 2017).

### 4.16 Immune cells trigger inflammatory and apoptosis processes post SCI

Inflammatory responses post SCI are induced by peripheral immune cells, and activated glial cells that proliferate and migrate to the lesions after SCI (Tian et al., 2007). Extensive inflammatory responses may result in apoptosis of oligodendrocytes and neurons and scar formation, ultimately causing dysfunction of neuronal cells. For instance, T cells play a critical role in activating macrophages, which can mount immune responses. Activated myelin basic protein (MBP)-reactive T cells may cause transient paralysis and neuroinflammation after SCI. The proportion of MBP-reactive T cells increases after injury, which reach infiltration levels that approximate those identified in patients with multiple sclerosis (Zhang et al., 2012). Although previous evidence indicated that B cells were pathological after SCI, the pathogenic capacity of SCI-triggered B cells still remained to be investigated (Genovese et al., 2006). Direct links between SCI and activation of peripheral lymphocytes have been verified. Once lymphocytes migrate into the SCI lesions, these cells indefinitely persist. The relative prevalence of B and T cells increases in injured spinal cord tissues of SCI mice through more than 9 weeks after SCI (Thuret et al., 2006). Neutrophils and macrophages have also been implicated in the tissue damage and expand of lesion sites. Macrophages may induce the inflammatory responses and secondary injuries, partially through the release of cytokines (Zhou et al., 2009). Inhibiting abnormal inflammatory responses may suppress tissue scarring, neuronal apoptosis, and neurological impairment post SCI.

### 4.17 Implications for designing multi-pronged anti-apoptotic therapies for treating SCI

Multiple drugs, hyperbaric oxygen, surgery, rehabilitation, as well as other therapeutic strategies have been utilized for treating SCI and its sequelae for over 50 years in clinical practice, whereas the therapeutic efficacies of these methods are not that satisfactory. Suppressing neuronal apoptosis is significant in the functional recovery after SCI (Ramer et al., 2014).

Currently, the most widely used therapy for acute SCI is the treatment with high-dose steroid methylprednisolone (Liu et al., 2020), which is mainly aimed at the acute secondary SCI process. Its efficacy has been repeatedly validated in animal SCI models and in two major clinical trials of Homo sapiens. The mechanism of action is considered to be its capacity for antagonizing lipid peroxidation, therefore preserving membrane integrity and reducing calcium influx and cell damage. It might also have other functions, such as retarding the influx of immunocytes and therefore repressing the effects of cytokine production, which can influence injuries to DNA induced by reactive oxygen species liberated by peroxidation (Kaptanoglu et al., 2000). Hence, it has potential effects on apoptosis and acute secondary processes. In SCI rats, intraperitoneal injection with dexamethasone post SCI significantly inhibits apoptosis in both glial cells and neuronal cells (Zurita et al., 2002), and this effect may be mediated via inhibiting TNF-α and NF-κB (Zirak et al., 2021). Although methylprednisolone has been widely utilized in SCI treatments, it exhibited deleterious impacts on spinal cord tissues.

A critical principle in designing the future interventions is to target therapies to core steps in SCI cascades, which may allow reduced side effects and improved specificity of action. Active cell death regulated by various receptors and activated intracellular signaling pathways which last for weeks post SCI indicates a novel treatment window for SCI. A potential significant therapeutic target might be the oligodendrocytes that die in response to Wallerian degeneration (Liu et al., 1997). A previous study (Liu et al., 1997) revealed a significant uptrend in the spared rim of tissues, and an impressive enhancement of functional recovery, by utilizing cycloheximide therapies. Though there is no definitive evidence demonstrating cycloheximide effects are induced by a decrease of apoptosis and a sparing of myelin and oligodendrocytes, to a certain extent, these findings support for the possible significance of oligodendroglial (long-term) and neuronal (short-term) apoptosis as the main effectors of secondary damage. Moreover, decrease of destruction by cycloheximide indicates treatments aimed at apoptosis may be clinically useful. Recent evidence has suggested possible therapeutic benefit of peptides that inhibit the activity of interleukin-1-beta converting enzyme-like proteases (caspases) in an ischemia SCI model (Abbaszadeh et al., 2020). Caspases play a critical role in the effector system of apoptosis (Keane et al., 2001). Therapeutic strategies should be aimed at inhibiting inflammatory cytokine-induced apoptosis, at suppressing intracellular effectors of apoptosis including the caspase pathway, and at promoting the endogenous cellular mechanisms that suppress apoptosis. Treatments that combine enhancement of neuronal repair mechanisms with control of apoptosis may be specifically effective in promoting functional recovery after SCI.

As naturally-derived compounds, phytochemicals have been identified in various functional foods with protective and anti-apoptosis effects against oxidative stress and neuroinflammation in SCI (Kim J. et al., 2010; Zhang et al., 2017). These compounds have been demonstrated to show ameliorative effects on apoptosis-related pathways (Martau et al., 2023). Polyphenols, amongst phytochemicals, terpenes/terpenoids, and alkaloids have promising modulatory effects on apoptosis after SCI (Mandel and Youdim, 2004; Lu et al., 2013).

Transplantation of MSCs, such as bone marrow mesenchymal stem cells (BMSCs), embryonic stem cells, neural stem cells, Schwann cells, and human umbilical cord blood cells has been considered as a possible therapy for SCI (Ruff et al., 2012; Zhou et al., 2012). Nevertheless, the efficacy of MSC transplantation therapy is largely blunted in clinical applications due to disadvantages including genetic variation, immunological rejection, the unsatisfactory cell survival rate, and complex operation process (Urdzíkova et al., 2006; Herberts et al., 2011; Yazdani et al., 2012; Zhang J. Y. et al., 2015). Treatment effects of stem-cell transplantation treatment in SCI have been validated in some studies, in which paracrine mechanisms in stem cells function as a critical role (Camussi et al., 2013). In recent years, many researches have demonstrated exosomes generated from multiple cell types have positive effects on the neuronal recovery after SCI (Huang et al., 2017; Tao et al., 2017; Wang et al., 2018). An increasing number of studies considered exosome transplantation as a possible alternative to transplantation of MSCs (Roccaro et al., 2013; Lener et al., 2015). A previous study revealed that BMSCs-Exosomes can effectively activate the Wnt/β-catenin pathway, which may suppress neural apoptotic cell-death induced by lipopolysaccharide (Li et al., 2019), indicating that transplantation of BMSCs-Exosomes might be a potential anti-apoptotic treatment for SCI via activating Wnt/β-catenin pathway.

However, there is still several limitations to this study. Firstly, we only focused on literatures from the public data source. Integrating the findings with sources from other datasets (e.g., Scopus and PubMed) may have been better. It was worth emphasizing that WOS was most commonly used in bibliometric analysis, and most bibliometric tools could obtain valuable information from the database. Secondly, as apoptosis and SCI has been a consistently evolving research field in recent decades, this study might have underestimated documents recently published in high-quality journals, which exhibited low citation number during the period. Thirdly, results obtained based on the bibliometric analysis might be different from the real-world research conditions. Fourthly, we only analyzed the key words occurring more than 10 times in the field, which may overlook some recent key words. Finally, the study period of our work covered a period of time when there was less literature published, which may cause bias to some extent.

## 5 Conclusion

This bibliometric study reviewed the development made in the past 30 years in apoptosis and SCI, which revealed the hot topics and research frontiers that may inform future directions. Our findings indicated that the USA and China were major contributors to the field of apoptosis and SCI. Cuzzocrea S and Mazzon E were the most influential authors, who have published significantly impacted researches on apoptosis and SCI. Apoptosis after primary SCI may have important effects on secondary SCI mechanisms, which cause the ultimate neurological deficits, whereas the molecular and cellular mechanisms require in-depth exploration. Inhibiting apoptosis plays a crucial role in improving complications after SCI. Caspases, Bax/Bcl-2, TNF-α, and JAK/STAT show interconnections with various pro-inflammatory pathways, including PI3K/Akt/mTOR, ERK/MAPK, glutamatergic pathways in apoptosis post SCI. A better understanding of the apoptosis-related pathways may inform significant therapeutic methods. The complexity of apoptosis-related pathways and their interactions in SCI urge the need for identifying multi-target agents as well as multi-pronged therapies with more tolerability and safety. It is worth emphasizing that the complexity of SCI cannot be limited to a single physio-pathological mechanism or to activation or suppression of a single type of cell-death. Hence, combination treatments targeting different programmed cell death modalities may be more effective strategies for SCI. More efforts on translating current advances into therapeutic strategies to activate or inhibit cell-death modalities is urgently needed in the field of apoptosis and SCI.

## Data availability statement

The original contributions presented in the study are included in the article/Supplementary material, further inquiries can be directed to the corresponding author.

## Author contributions

SW: Conceptualization, Data curation, Formal analysis, Investigation, Methodology, Project administration, Resources, Software, Supervision, Validation, Visualization, Writing—original draft, Writing—review and editing. LC: Conceptualization, Data curation, Formal analysis, Funding acquisition, Investigation, Methodology, Project administration, Resources, Software, Supervision, Validation, Visualization, Writing—review and editing.

## Footnotes

1
www.r-project.org

## Supplementary material

Supplementary Figure 1 Flowchart of inclusion and exclusion criteria for relevant documents.

Supplementary Figure 2 **(A)** The top 10 most cited countries. Diameter and color darkness of the node was in proportion to the number of documents published by the country or region. **(B)** Corresponding author’s countries. SCP: single country publication; MCP: multiple countries publications. **(C)** The frequency distribution of scientific productivity. **(D)** The top 10 authors’ production over time. Red line represented the length of years when the author published related articles. Color darkness and size of the nodes was in proportion to the number of documents published by the author.

Supplementary Figure 3 Analysis of the core sources. The horizontal axis was labeled sources related to apoptosis and spinal cord injury. The vertical axis represented the number of articles published on each source.

Supplementary Figure 4 **(A)** The top 10 most local cited references. Size and darkness of the nodes represented the citation number of each reference. **(B)** Sankey diagram of cited references, authors, and author’s key words.

Supplementary Figure 5 **(A)** Key word tree of key words selected from Keyword Plus. The size of Square represented key word’s occurrence compared with the whole occurrences. **(B)** Key word cloud diagram. The size of words represented the occurrence frequency of the relative key words.

Supplementary Figure 6 Analysis of trend topics related to apoptosis and spinal cord injury during to past 30 years. **(A)** Topic dendrogram. Topics that shared close relationship were closer on the dendrogram. Two main branches were labeled blue and red, respectively. **(B)** Conceptual structure map of themes. Topics that shared close relationship were closer on the map.
