# Supplementary figures and images for: The role of apoptosis in spinal cord injury: a bibliometric analysis from 1994 to 2023
Source: Front Cell Neurosci. 2024 Jan 16;17:1334092. doi: 10.3389/fncel.2023.1334092 (PMC10825042; doi:10.3389/fncel.2023.1334092)

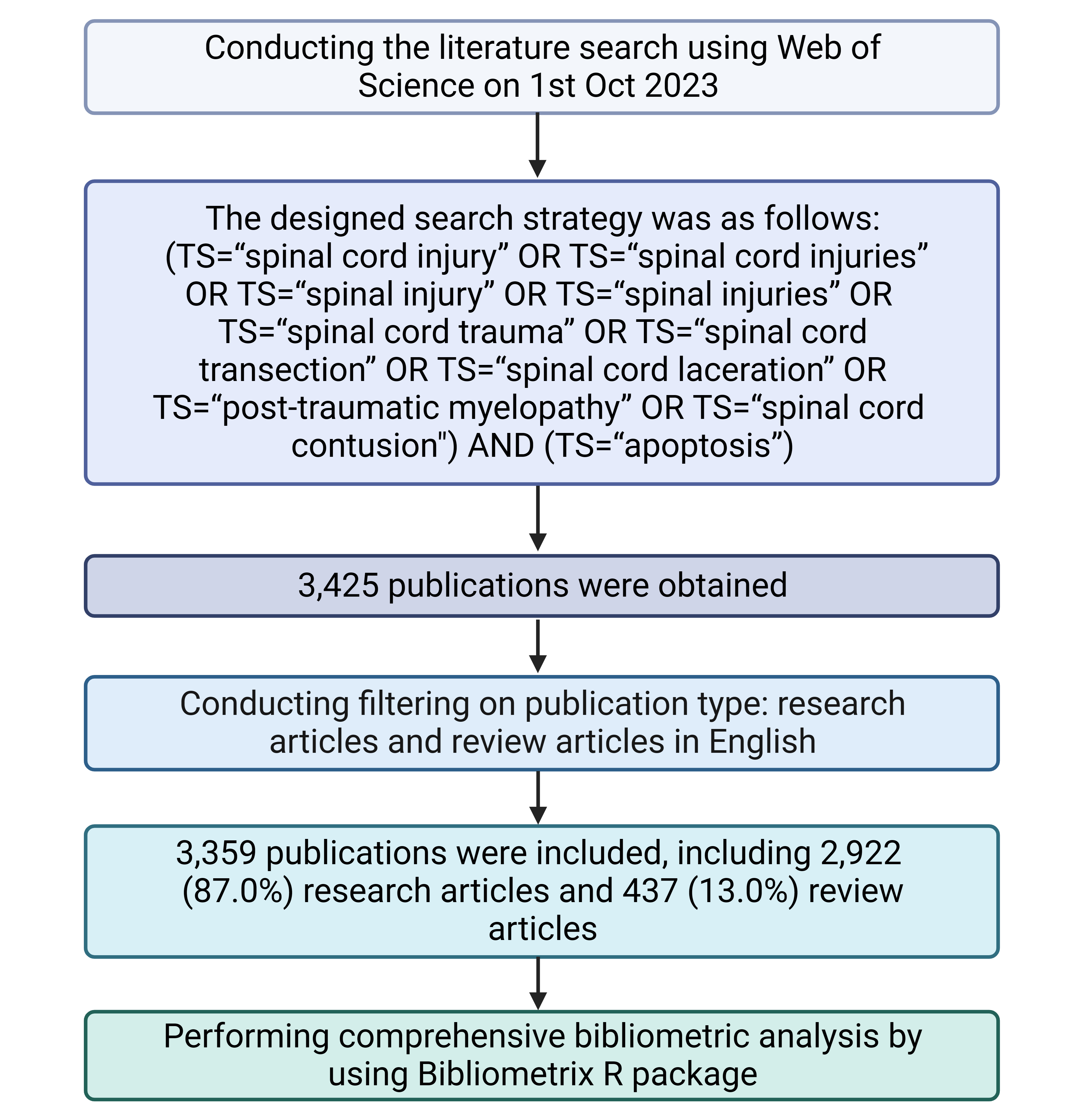

Supplement: Supplementary Figure 1 — Flowchart of inclusion and exclusion criteria for relevant documents. [file Image_1.TIFF]

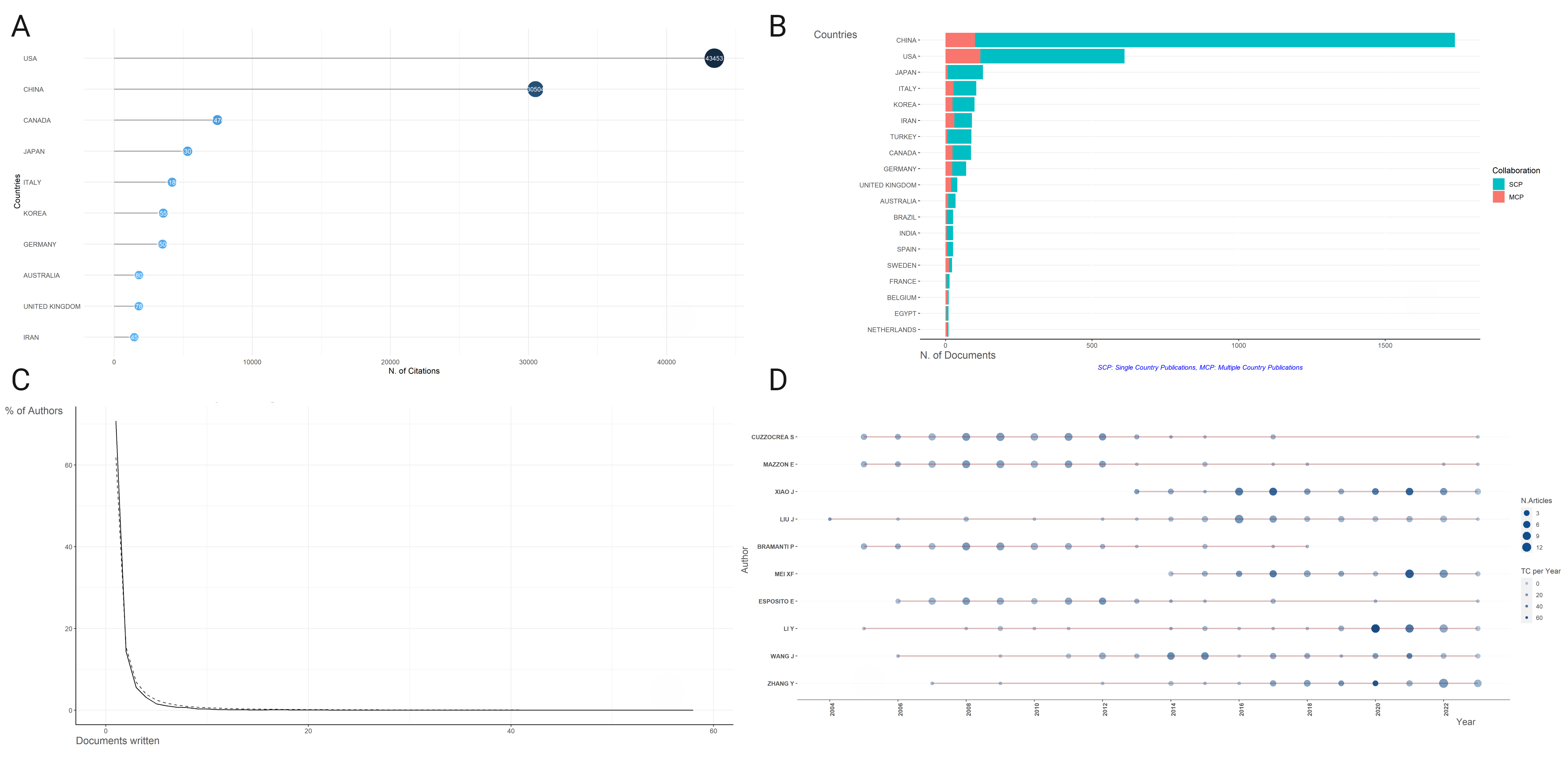

Supplement: Supplementary Figure 2 — (A) The top 10 most cited countries. Diameter and color darkness of the node was in proportion to the number of documents published by the country or region. (B) Corresponding author’s countries. SCP: single country publication; MCP: multiple countries publications. (C) The frequency distribution of scientific productivity. (D) The top 10 authors’ production over time. Red line represented the length of years when the author published related articles. Color darkness and size of the nodes was in proportion to the number of documents published by the author. [file Image_2.TIFF]

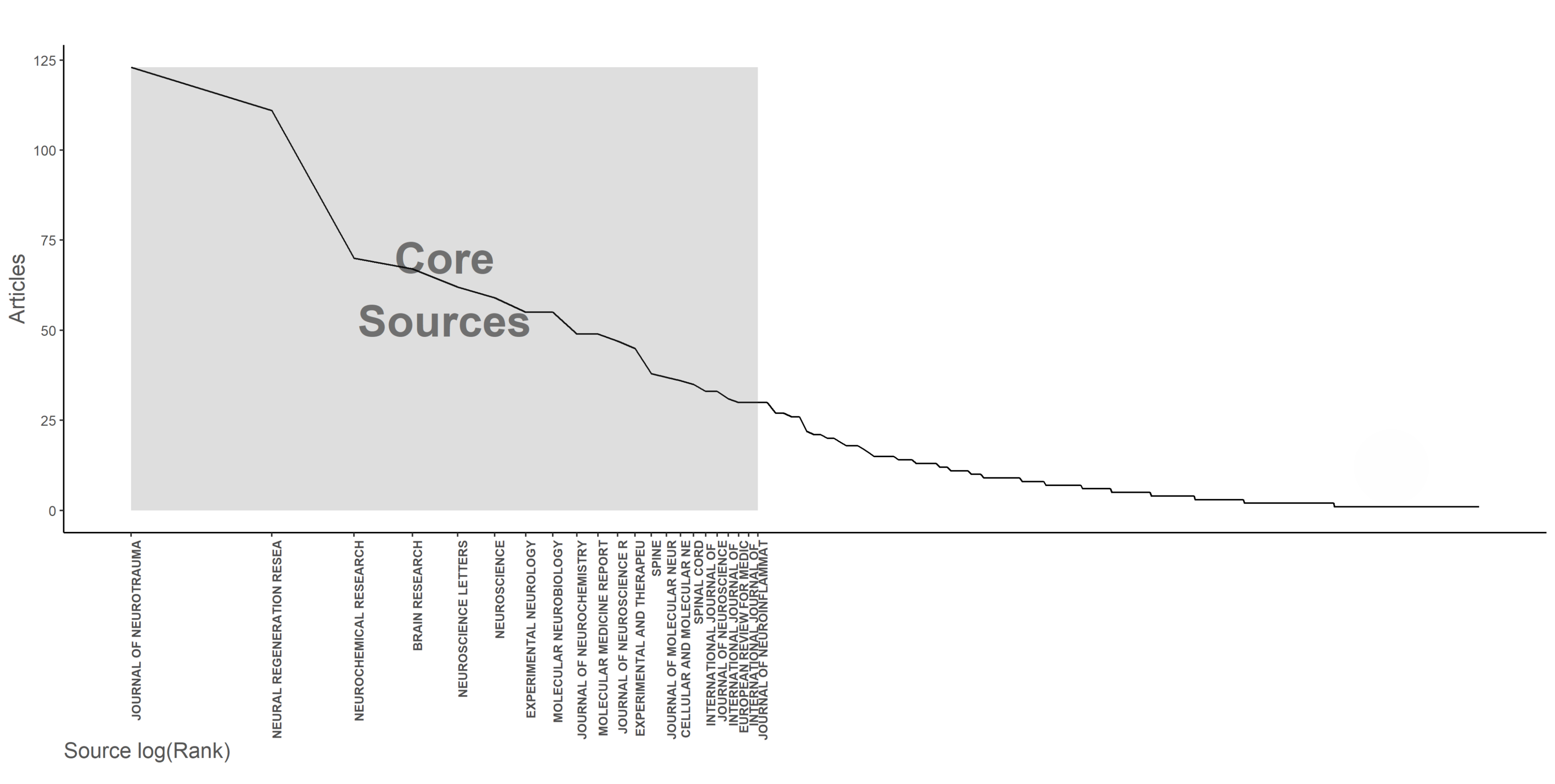

Supplement: Supplementary Figure 3 — Analysis of the core sources. The horizontal axis was labeled sources related to apoptosis and spinal cord injury. The vertical axis represented the number of articles published on each source. [file Image_3.TIFF]

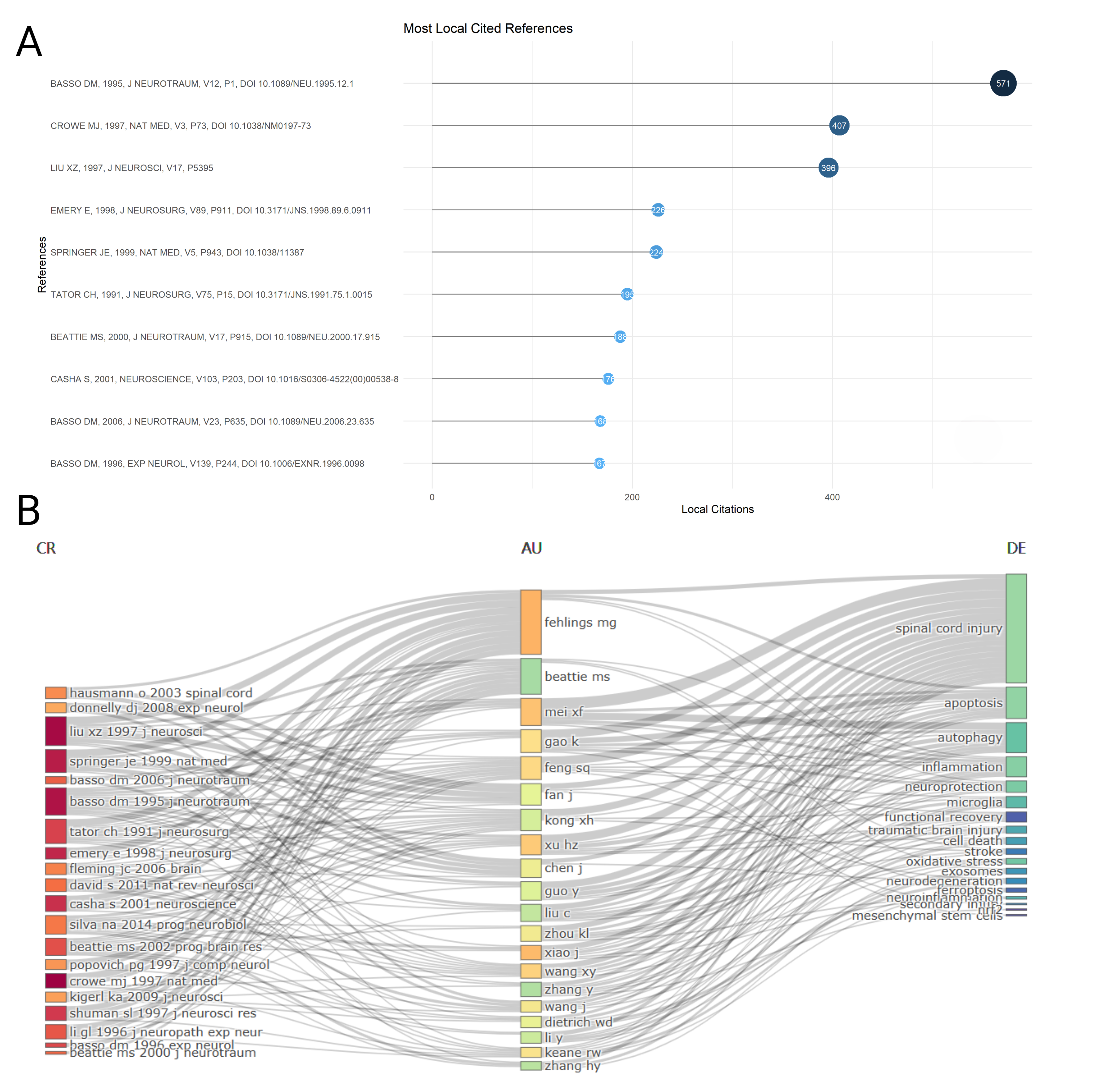

Supplement: Supplementary Figure 4 — (A) The top 10 most local cited references. Size and darkness of the nodes represented the citation number of each reference. (B) Sankey diagram of cited references, authors, and author’s key words. [file Image_4.TIFF]

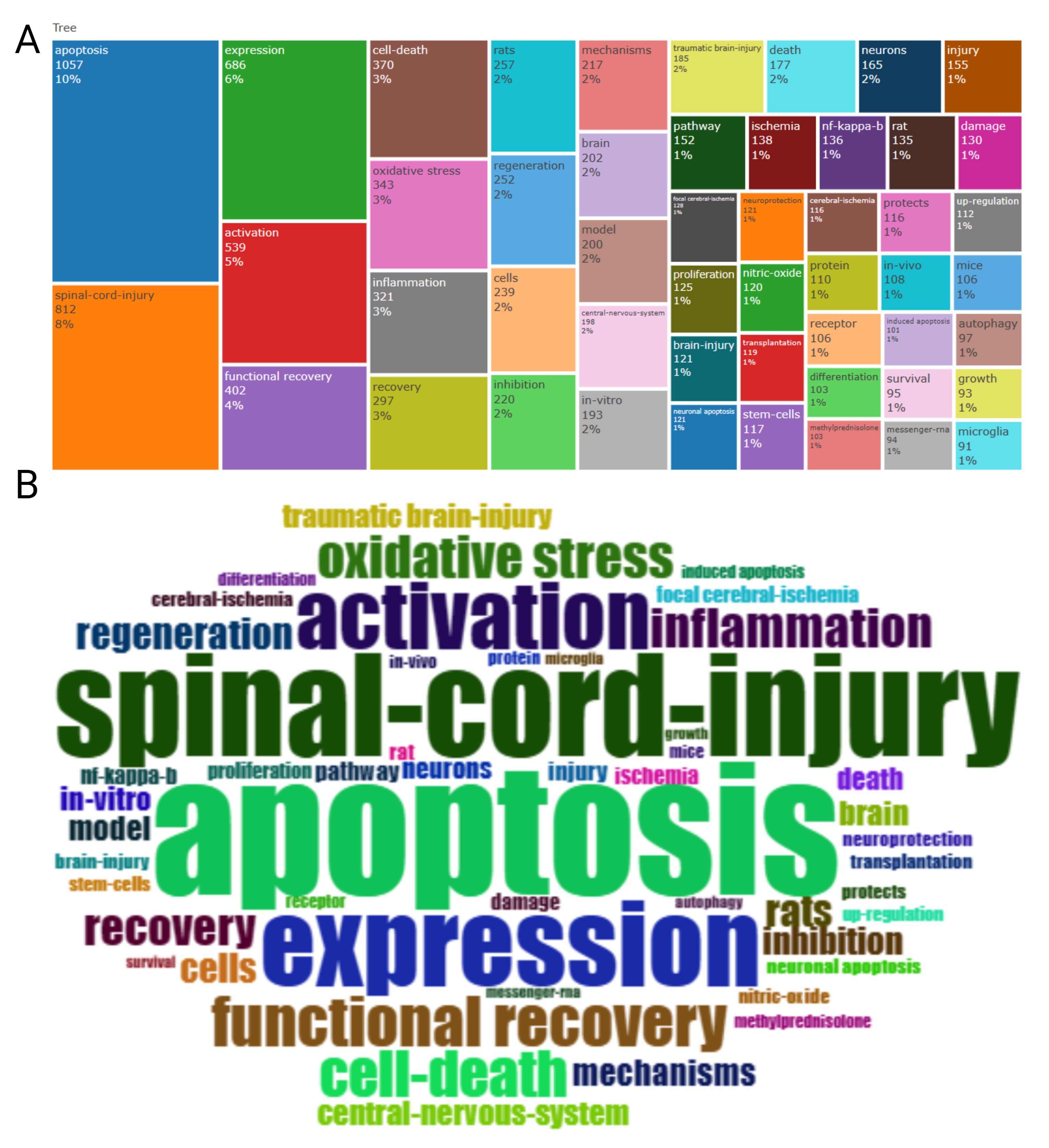

Supplement: Supplementary Figure 5 — (A) Key word tree of key words selected from Keyword Plus. The size of Square represented key word’s occurrence compared with the whole occurrences. (B) Key word cloud diagram. The size of words represented the occurrence frequency of the relative key words. [file Image_5.TIFF]

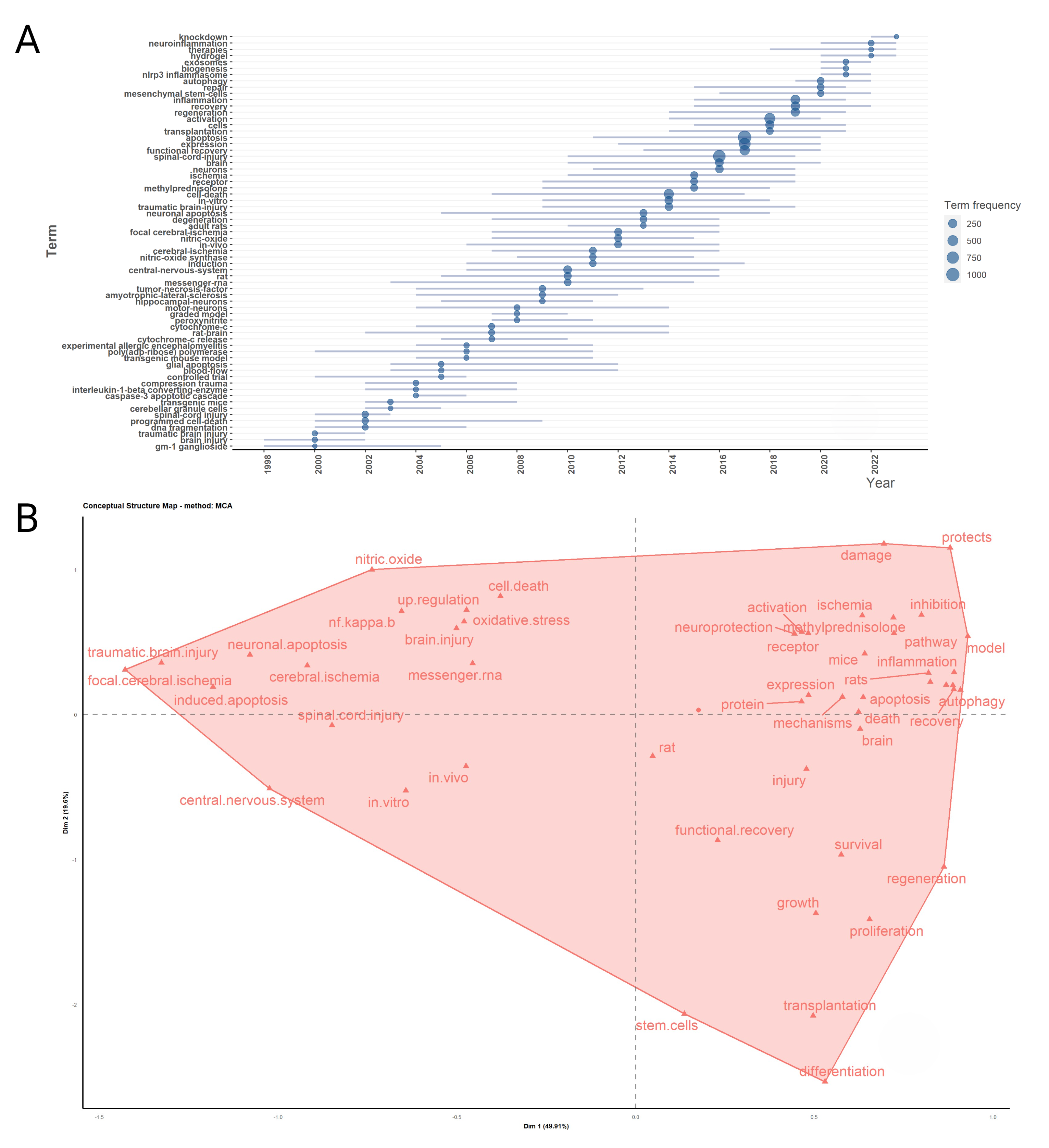

Supplement: Supplementary Figure 6 — Analysis of trend topics related to apoptosis and spinal cord injury during to past 30 years. (A) Topic dendrogram. Topics that shared close relationship were closer on the dendrogram. Two main branches were labeled blue and red, respectively. (B) Conceptual structure map of themes. Topics that shared close relationship were closer on the map. [file Image_6.TIFF]
